# In vitro selection and analysis of SARS-CoV-2 nirmatrelvir resistance mutations contributing to clinical virus resistance surveillance

**DOI:** 10.1126/sciadv.adl4013

**Published:** 2024-07-24

**Authors:** Yuao Zhu, Irina Yurgelonis, Stephen Noell, Qingyi Yang, Shunjie Guan, Zhenghui Li, Li Hao, Hussin Rothan, Devendra K. Rai, Patricia McMonagle, Mary Lynn Baniecki, Samantha E. Greasley, Olga Plotnikova, Jonathan Lee, Jennifer A. Nicki, RoseAnn Ferre, Laura J. Byrnes, Wei Liu, Timothy K. Craig, Claire M. Steppan, Paul Liberator, Holly D. Soares, Charlotte M. N. Allerton, Annaliesa S. Anderson, Rhonda D. Cardin

**Affiliations:** ^1^ Pfizer Worldwide Research, Development & Medical, Pearl River, NY 10965, USA.; ^2^ Pfizer Worldwide Research, Development & Medical, Groton, CT 06340, USA.; ^3^ Pfizer Worldwide Research, Development & Medical, Cambridge MA 02139, USA.; ^4^ Pfizer Worldwide Research, Development & Medical, La Jolla, CA 92121, USA.

## Abstract

To facilitate the detection and management of potential clinical antiviral resistance, in vitro selection of drug-resistant severe acute respiratory syndrome coronavirus 2 (SARS-CoV-2) against the virus M^pro^ inhibitor nirmatrelvir (Paxlovid active component) was conducted. Six M^pro^ mutation patterns containing T304I alone or in combination with T21I, L50F, T135I, S144A, or A173V emerged, with A173V+T304I and T21I+S144A+T304I mutations showing >20-fold resistance each. Biochemical analyses indicated inhibition constant shifts aligned to antiviral results, with S144A and A173V each markedly reducing nirmatrelvir inhibition and M^pro^ activity. SARS-CoV-2 surveillance revealed that in vitro resistance–associated mutations from our studies and those reported in the literature were rarely detected in the Global Initiative on Sharing All Influenza Data database. In the Paxlovid Evaluation of Protease Inhibition for COVID-19 in High-Risk Patients trial, E166V was the only emergent resistance mutation, observed in three Paxlovid-treated patients, none of whom experienced COVID-19–related hospitalization or death.

## Teaser

SARS-CoV-2 nirmatrelvir in vitro resistance analyses guide the surveillance of circulating virus and clinical virology assessment.

## INTRODUCTION

Severe acute respiratory syndrome coronavirus 2 (SARS-CoV-2) encodes a main protease (M^pro^, nsp5, 3CL^pro^) that is essential for virus replication (*1*, *2*). M^pro^ cleaves coronavirus polyprotein 1a and 1ab at 11 junctions with a conserved Gln residue at the P1 site, a feature with no known human analogs (*3*–*5*). The essential function in coronavirus replication, together with the absence of closely related homologs in humans, identifies M^pro^ as an attractive antiviral drug target (*6*). The SARS-CoV-2 M^pro^ inhibitor, nirmatrelvir, was found and developed as the oral COVID-19 treatment Paxlovid (nirmatrelvir/ritonavir) (*7*). In the phase 2/3 EPIC-HR (Evaluation of Protease Inhibition for COVID-19 in High-Risk Patients) clinical study (NCT04960202), Paxlovid reduced the incidence of severe COVID-19 (hospitalization or death) by 86% (*8*) in high-risk patients when treatment started within 5 days of symptom onset (*9*). Furthermore, in large-scale real-world treatment studies, Paxlovid significantly reduced COVID-19–related hospitalization or death in older indicated patients infected with Omicron variants (*10*–*12*).

Antiviral treatment can lead to selection of drug-resistant viruses resulting in loss of efficacy, as evidenced in numerous antiviral therapeutics and in patients with COVID-19 treated with remdesivir (*13*–*19*). In line with the U.S. National Institutes of Health (*20*) and Food and Drug Administration (*21*) guidelines, we undertook in vitro passaging of SARS-CoV-2 in the presence of nirmatrelvir to identify potential drug-resistant viruses with mutations in the target M^pro^ gene. The identification and characterization of these drug-resistant mutant viruses could assist in the early detection and management of potential resistance to Paxlovid treatment. In this study, nirmatrelvir-resistant viruses were selected in cell cultures and evaluated for drug resistance in antiviral assays. Biochemical and structural biology analyses were conducted to gain insight into their possible mechanisms of resistance. Furthermore, an analysis of the prevalence of the M^pro^ mutations identified in this study and from in vitro resistance selection studies reported in the literature (*22*–*24*), as well as their potential impact on treatment outcome in EPIC-HR clinical trial participants (*9*), was undertaken.

## RESULTS

**Fig. 1.**
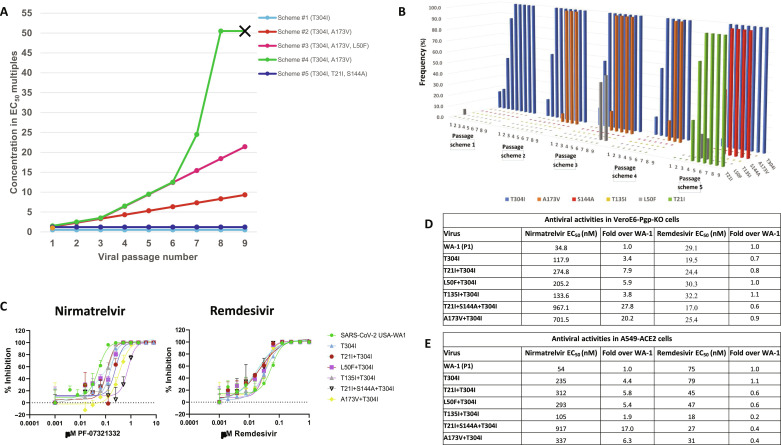
Selection of SARS-CoV-2 with reduced susceptibility to nirmatrelvir. (**A**) Virus selection schemes. Five virus passage schemes with nirmatrelvir concentration indicated as EC_50_ multiples, as well as the dominant M^pro^ mutations in each scheme are shown. (**B**) Selection of M^pro^ mutation in each virus passage scheme and change in frequency of each mutation along virus passages in virus populations. Frequencies of each of the six individual M^pro^ mutations are indicated. (**C**) Examples of dose-response curves of the six identified and plaque purified M^pro^ mutant viruses against nirmatrelvir or remdesivir in VeroE6-Pgp-KO cells. (**D**) Antiviral activities of nirmatrelvir and remdesivir each against the six M^pro^ mutant viruses and WT virus in VeroE6-Pgp-KO cells. EC_50_ values and fold change from WT virus are shown. (**E**) Antiviral activities of nirmatrelvir and remdesivir in A549-ACE2 cells.

Nirmatrelvir, the active component of Paxlovid, exhibits potent antiviral activity against SARS-CoV-2; however, it is a substrate of the efflux pump P-gp [P-glycoprotein; MDR1 (multidrug resistance 1)], leading to lower intracellular nirmatrelvir concentrations in cells overexpressing Pgp (*7*). Therefore, a recently established VeroE6 cell line containing the *pgp* gene knockout (VeroE6-Pgp-KO) was used for the in vitro selection of resistant viruses by passaging virus in the presence of nirmatrelvir (*25*). Nirmatrelvir antiviral activity against SARS-CoV-2 was first evaluated in this cell line using a cytopathic effect (CPE)–based assay, which resulted in estimated 50 and 90% effective concentration (EC_50_ and EC_90_) values of 0.15 and 0.37 μM, respectively. For drug resistance selection, the SARS-CoV-2 WA-1 strain was serially passaged in five different passage and drug treatment schemes including nirmatrelvir treatment at constant concentrations of 1× EC_50_ and EC_90_ levels, as well as stepwise increasing drug concentrations (Fig. 1A). Viruses harvested from each passage were analyzed with next-generation sequencing (NGS). The dominant mutations in M^pro^ that emerged from each scheme are indicated in Fig. 1A, and the evolution of the selected M^pro^ mutations and frequencies in each passage scheme are summarized in Fig. 1B. T304I became detectable (>5% frequency) after the first passage at 1×EC_50_ or second passage at 1×EC_90_ nirmatrelvir levels in all passage schemes (Fig. 1B). T304I was also the only substitution identified in scheme 1 when the virus was passaged at a constant concentration of 0.15 μM nirmatrelvir (1×EC_50_) over nine passages. When resistance selection was carried out at escalating nirmatrelvir concentrations in schemes 2, 3, and 4, an additional A173V mutation appeared after five or six passages at concentrations of 0.9 or 1.3 μM (~5%) and further enriched to nearly 100% at T304I and A173V sites over subsequent passages (Fig. 1B). As nirmatrelvir concentration increased to 7.3 μM (50×EC_50_) in scheme 4 for two passages, no virus induced CPE or viral RNA could be detected, indicating a complete suppression of replication of A173V+T304I mutant virus at this drug concentration (Fig. 1A). In scheme 5, selection at a constant EC_90_ nirmatrelvir level led to the emergence of T21I, L50F, T135I, S144A, and A191V in addition to T304I at varying frequencies and combinations following passage 4 (Fig. 1B), as revealed by virus plaques containing T21I+T304I, L50F+T304I, and T135I+T304I, which were then overtaken by the T21I+S144A+T304I triple mutation as the dominant species (Fig. 1, A and B). The emergence of the triple mutation presumably reflects a dynamic interplay of virus quasi-species evolution at a constant suboptimal drug pressure when virus replication “space” is enhanced through passages at a low multiplicity of infection (MOI). As a control, the parent virus SARS-CoV-2 USA-WA1 passaged in 0.3% dimethyl sulfoxide (DMSO) had no mutations identified in M^pro^ through passage 10.

To purify the mutant viruses, 88 virus plaques were isolated and sequenced, which led to the isolation of mutant viruses containing six mutation patterns of T304I, T21I+T304I, L50F+T304I, T135I+T304I, A173V+T304I, T21I+S144A+T304I. These mutant SARS-CoV-2 isolates were each tested for susceptibility to nirmatrelvir and remdesivir in VeroE6-Pgp-KO cells, the cell line from which they were selected. An example dose-response curve for each mutant is shown in Fig. 1C, with the mean EC_50_ and EC_90_ values as well as fold changes from the wild-type (WT) parent indicated in Fig. 1D. The T304I mutation alone reduced nirmatrelvir susceptibility by 3.4-fold, while addition of T21I, L50F, or T135I each slightly further reduced nirmatrelvir sensitivity, resulting in 3.8- to 7.9-fold reduced susceptibilities (Fig. 1D). The triple mutant T21I+S144A+T304I conferred 27.8-fold resistance, whereas the A173V+T304I mutant that emerged in all three selection schemes with escalating nirmatrelvir concentrations led to 20.2-fold resistance (Fig. 1D). All the mutants remained sensitive to remdesivir at levels similar to the WT virus (Fig. 1D). To determine whether the observed antiviral activities were not cell type specific, the six mutant viruses were also tested in A549-ACE2 cells, a lung carcinoma cell line. The overall results were similar to that in VeroE6-Pgp-KO cells, with the T21I+S144A+T304I and A173V+T304I each conferring slightly less fold resistance at 17.1- and 6.3-fold, respectively (Fig. 1E). All six mutant viruses remained sensitive to remdesivir (Fig. 1E).

Several other concurrent mutations occurred elsewhere in the genomes of the six mutant virus quasi-species populations (table S1). Two mutations, one in nsp13 helicase (S468L) and one in orf3a (I37V), were common to the T304I, T21I+T304I, L50F+T304I, T135I+T304I, and T21I+S144A+T304I mutant viruses. These mutations were not detected in the control passaged virus. Because these five M^pro^ mutant viruses were selected from the same passage scheme 5, and the T304I emerged first, this finding is consistent with a stepwise selection of a combination of mutations, reminiscent of the protease inhibitor–resistant virus selection observed in HIV protease inhibitor monotherapy (*26*). The T21I+T304I virus acquired a nsp14 exonuclease I87V mutation, which existed in the T21I+S144A+T304I triple mutant that appeared in later passages, further supporting a stepwise selection (table S1). From a separate selection scheme, an A173V+T304I mutant had a different nsp13 K94N mutation as well as an nsp3 N1543K mutation in the ubiquitin-like (UBL)-2 region (table S1).

The nsp13 encodes the virus helicase that is essential for virus RNA replication, which may also inhibit type 1 interferon production (*27*) and/or suppress interferon signaling by perturbing Janus kinase 1 phosphorylation of signal transducer and activator of transcription 1 (*28*). Orf3a encodes a viroporin able to function as an ion channel that may promote virus release (*29*), and the nsp3 UBL-2 region is implicated in the innate immune response (*30*). The persistence of the nsp13 and orf3a mutations with passaging is consistent with a potential role in mutant virus replication in vitro.

**Fig. 2.**
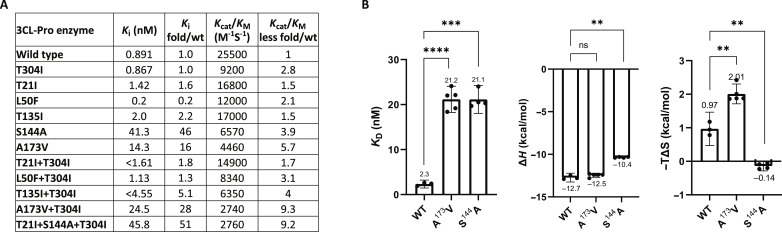
Biochemical evaluation of identified mutations. (**A**) Biochemical analysis of M^pro^ containing each of the indicated mutations. Nirmatrelvir enzymatic inhibition indicated as *K*_i_, and *K*_i_ over that of the WT are included. Enzyme catalytic activity is indicated as *k*_cat_/*K*_M_ for each mutant M^pro^, and fold less than WT. (**B**) ITC of purified M^pro^ WT and mutants. Comparison of thermodynamic properties (*K*_D_, ΔH, and −*T*Δ*S*) of nirmatrelvir binding to WT versus mutant M^pro^ proteins. Unpaired *t* test used for statistical analysis, ns, not significant, ***P < 0.001, ****P < 0.0001.

To evaluate whether the six M^pro^ mutation patterns as well as each of the single mutations affected M^pro^ enzyme activity and susceptibility to nirmatrelvir, each mutant enzyme was generated and analyzed in a biochemical assay (*31*). The catalytic efficiency (*k*_cat_/*K*_M_) and inhibition constant (*K*_i_) values, as well as the fold changes over WT, are summarized in Fig. 2A. Single mutations T304I, T21I, L50F, and T135I had minimum impact on nirmatrelvir *K*_i_ values, while the S144A and A173V mutations led to 46- or 16-fold increases in *K*_i_ over WT enzyme, respectively (Fig. 2A). Because the S144A and A173V mutations each led to significant *K*_i_ increases, these two M^pro^ mutants were further analyzed by isothermal titration calorimetry (ITC) (*32*). The *K*_D_ values obtained for nirmatrelvir to the A173V and S144A mutations were significantly higher than that of WT M^pro^ (Fig. 2B), with titration curves summarized in fig. S1. The ITC experiment showed that the binding potency loss in the A173V mutant compared to WT is due to an entropic effect, while the binding potency loss in the S144A mutation is primarily due to changes in the enthalpic contribution to binding (Fig. 2B). These biochemical data suggested that S144A and A173V were each the main contributors to the changes in *K*_i_ values of the T21I+S144A+T304I and A173V+T304I mutant enzymes (Fig. 2A) as well as the decreased sensitivities to nirmatrelvir of the corresponding mutant viruses (Fig. 1, D and E). The double-mutant enzymes T21I+T304I, L50F+T304I, and T135I+T304I either had no impact on *K*_i_ or had a lower fold change (Fig. 2A), consistent with the lower fold resistance of the corresponding mutant viruses against nirmatrelvir in culture (Fig. 1, D and E). The T21I+S144A+T304I and A173V+T304I mutant M^pro^ enzymes showed more than ninefold reduction in catalytic efficiency (*k*_cat_/*K*_M_) (Fig. 2A), implying a possible reduced virus fitness if these were the only mutations in the virus genome. The A173V and S144A mutant enzymes were also affected, with 5.7- and 3.9-fold reductions in *k*_cat_/*K*_M_, respectively (Fig. 2A). Other mutation patterns identified in resistance selection led to 1.7- to 4-fold reductions in *k*_cat_/*K*_M_ (Fig. 2A). These data are consistent with a need for adaptive mutations elsewhere in virus genomes for efficient replication in the tested cell lines, some of which could be the nsp13, nsp3, nsp14, and orf3a mutations described in table S1.

**Fig. 3.**
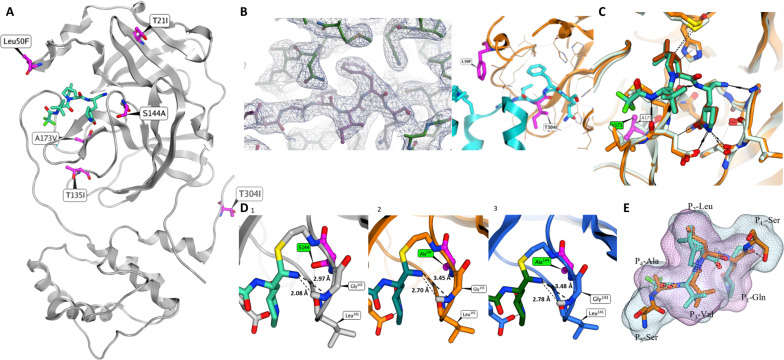
Structural modeling of mutant M^pro^ enzymes. (**A**) Location of the six selected individual mutations in M^pro^ structure. (**B**) (1 and 2) X-ray structure of T304I/L50F double-mutant apo M^pro^ (1) enzyme electron density of at the catalytic site. The C terminus is colored in pink. (2). Catalytic pocket with M^pro^ C terminus bound. L50F and T304I are labeled in purple. (**C**) Superposition of x-ray crystallography structures of SARS-COV2-M^pro^, WT (pdb 7RFS), and T304I+A173V mutant with nirmatrelvir bound at active site. The T304I+A173V mutation is displayed in yellow. The WT M^pro^ protein is in silver. The A173V mutant is labeled and highlighted purple. (**D**) X-ray structure comparison of M^pro^ WT ang S144A with nirmatrelvir. (1) X-ray crystallography Structures of SARS-COV2-M^pro^, WT (pdb 7RFS) and (2) S144A with nirmatrelvir bound at active site. The T304I mutation is displayed in yellow. (3) Triple mutation T304I+T21I+S144A with nirmatrelvir bound at active site. The WT M^pro^ protein is in silver, S144A mutant is in yellow, and T304I+T21I+S144A triple mutant is displayed in dark blue. (**E**) Superposition of nirmatrelvir (pdb 7RFS) and the native nsp4/nsp5 substrate peptide (PDB 7DVP) at the active site domain. The molecular surface of nirmatrelvir (pink mesh) and fragment of nsp4/nsp5 peptide, SAVLQ-S, (blue mesh) are displayed.

To gain insight into the mechanism by which the mutations caused changes in viral sensitivity to nirmatrelvir, we undertook structural analyses (Fig. 3A). The crystal structures of the M^pro^ mutants with the greatest reductions in susceptibility to nirmatrelvir were determined in the presence or absence of nirmatrelvir, including S144A, A173V, A173V+T304I, T21I+S144A+T304I, T21I+T304I, and L50F+T304I (Fig. 3 and table S2). The T21I+T304I and the L50F+T304I double-mutant M^pro^ apo crystal structures showed that the enzyme C terminus was bound at the catalytic site, presumably preventing nirmatrelvir from binding (Fig. 3B). Molecular dynamics simulations (MDSs) were performed to study the impact of the T304I mutation on nirmatrelvir binding. MDS of the SARS-CoV2 M^pro^ with the canonical nsp5/nsp6 substrate peptide [Protein Data Bank (PDB), 7 MB6 and 7T8M) (*33*) compared to the peptide with the T304I mutation indicated that the T304I-mutated peptide substrate displayed a stronger interaction with the M^pro^ primarily because of increased van der Waals interaction (fig. S2A). The structural implications of the A173V and S144A mutations, which either as individual or combination mutations caused significant increases in the nirmatrelvir M^pro^ *K*_i_, were also studied (Fig. 2A). Crystal structures of A173V and the A173V+T304I double-mutant M^pro^ with nirmatrelvir did not display any major binding conformation differences. ITC experiments further revealed that the weaker binding of nirmatrelvir to the A173V mutant enzyme was primarily due to change in entropy, *T*Δ*S*, because the A173V M^pro^ bound with nirmatrelvir was more organized because of less atomic fluctuation than the WT M^pro^ with nirmatrelvir bound (Figs. 2B  and 3C and fig. S1). Dynamic differences were also observed in MDS (fig. S2B) between A173V and WT M^pro^. Overall, the cocrystal structure of the M^pro^ S144A mutant complexed with nirmatrelvir was similar to the WT M^pro^/nirmatrelvir complex (Fig. 3D). It was noted that the hydrogen bond interaction between adduct imine nitrogen and G143 backbone NH could be significantly weaker in the S144A mutant than in WT M^pro^ because the distance between the imine nitrogen and G143 NH hydrogen is 2.08 Å in the WT M^pro^, while the distance in the S144A and T21I+S144A+T304I mutants was 2.80 Å (*34*). This imine nitrogen to G143 NH distance difference was also observed in the MDS analysis (fig. S2C). In WT enzyme, the S144 side-chain hydroxyl group forms hydrogen bond interaction with the L141 backbone carbonyl group and stabilizes the loop. Because the S144A mutant lacks this hydrogen bond interaction, the loop comprising L141-S144 could be less structured compared to WT and consequently lead to the weaker hydrogen bond interaction between the adduct imine and G143 NH. This could explain the decreased enthalpic contribution in the S144A mutant enzyme binding as observed by ITC (Fig. 2B). Nirmatrelvir, as a peptido-mimetic inhibitor, shares high structural similarity to the M^pro^ substrate peptide. Its binding site adjusted shape overlap scores with peptide substrates nsp4/nsp5, nsp5/6, and nsp6/7 ranged from 86.6 to 91.4% (solvent adjusted) (Fig. 3E and table S3). This high degree of structural similarity may pose a challenge for the virus to generate single effective resistance mutations within the nirmatrelvir binding site without compromising its own enzyme activity and, hence, viral fitness, as was observed in HIV protease inhibitor primary resistance mutations (*35*). In comparison to nirmatrelvir, our previously studied M^pro^ inhibitor PF-00835231 (*36*), as well as S-217622 (ensitrelvir) (*37*), PF-00835231 showed lower adjusted overlap scores with M^pro^ peptide substrates (69.6 to 87.5%) while the adjusted overlap scores of S-217622 were substantially lower (61.3 to 65.1%) (table S3).

**Fig. 4.**
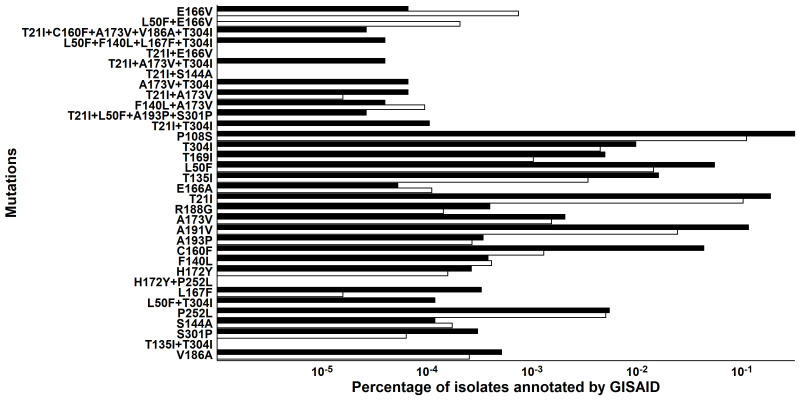
Prevalence of in vitro nirmatrelvir resistance associated M^pro^ mutations identified in GISAID database. M^pro^ mutations identified in the current study and recent publications present in the GISAID database, in which (7,585,338 SARS-CoV-2 sequence entries pre-EUA (black, 1 December 2019 to 21 December 2021) and 6,312,678 sequence entries post-EUA (white, 23 February 2022 to 31 January 2024) were included).

To assess the potential for clinical resistance to Paxlovid, a bioinformatic analysis of the clinical SARS-CoV-2 whole-genome sequences from the Global Initiative on Sharing All Influenza Data (GISAID) database (*38*) was used to determine whether the in vitro resistance mutations identified in this manuscript, as well as those reported from similar studies (*22*–*24*), were currently in circulation, and whether there were any changes in frequency before (pre-) or after (post-) the Paxlovid Emergency Use Authorization (EUA) was issued in December 2021. Results indicate that the M^pro^ resistance mutations that existed as individual or combination mutations were not abundant in the GISAID database and remained low post-Paxlovid EUA (Fig. 4).

To investigate whether any of the M^pro^ mutations selected in culture emerged during Paxlovid treatment or affected treatment outcome in the recent EPIC-HR (NCT04960202) Paxlovid clinical trial, we performed NGS analyses on patient nasal swab samples (D1, D3, D5, D10, and D14) with a sufficient viral RNA level. Of a total of 1038 participants in Paxlovid arm and 1053 in placebo arm who were randomized and took at least one dose of study intervention, baseline and/or postbaseline viral sequencing data were available from 765 Paxlovid and 757 placebo participants. Baseline as well as postbaseline emergent M^pro^ resistance associated mutations observed in EPIC-HR are summarized by treatment arm in Table 1.

**Table 1.** Detection and frequency (substitutions/RAS with amino acid frequency ≥10% were included) of the in vitro nirmatrelvir resistance associated M^pro^ mutations in Paxlovid- and placebo-treated patients in the EPIC-HR clinical trial.

	Baseline mutation		Emergent mutation	
M^pro^ mutation	Paxlovid (N* = 715)	Placebo (N* = 757)	Paxlovid (*N*† = 528)	Placebo (*N*† = 548)
T21I	0	0	0	0
L50F	1 (100%)	1 (100%)	0	0
P108S	1 (13%)	3 (100%, 100%, 100%)	0	1 (11%)
T135I	0	0	0	0
F140L	0	0	0	0
S144A	0	0	0	0
C160F	0	0	0	0
E166A	0	0	0	0
E166V	0	0	3 (24%, 90%, 94%)	0
L167F	0	0	0	0
T169I	0	0	0	0
H172Y	0	0	0	0
A173V	0	0	0	0
V186A	0	0	0	0
R188G	0	0	0	0
A191V	0	2 (99%, 51%)	0	0
A193P	0	0	0	0
P252L	0	0	0	0
S301P	0	0	0	0
T304I	0	0	1 (24%)	0
T21I+S144A	0	0	0	0
T21I+E166V	0	0	0	0
T21I+A173V	0	0	0	0
T21I+T304I	0	0	0	0
T21I+S144A+T304I	0	0	0	0
T21I+A173V+T304I	0	0	0	0
T21I+L50F+A193P+S301P	0	0	0	0
T21I+C160F+A173V+V186A+T304I	0	0	0	0
L50F+E166V	0	0	1 (L50F 100% + E166V 90%)	0
L50F+F140L+L167F+T304I	0	0	0	0
A173V+T304I	0	0	0	0
T135I+T304I	0	0	0	0
F140L+A173V	0	0	0	0
H172Y+P252L	0	0	0	0

*N represents the number of participants with available viral sequencing data at baseline by treatment group.

†*N* represents the number of participants with available viral sequencing data at baseline and one or more postbaseline visits.

**Fig. 5.**
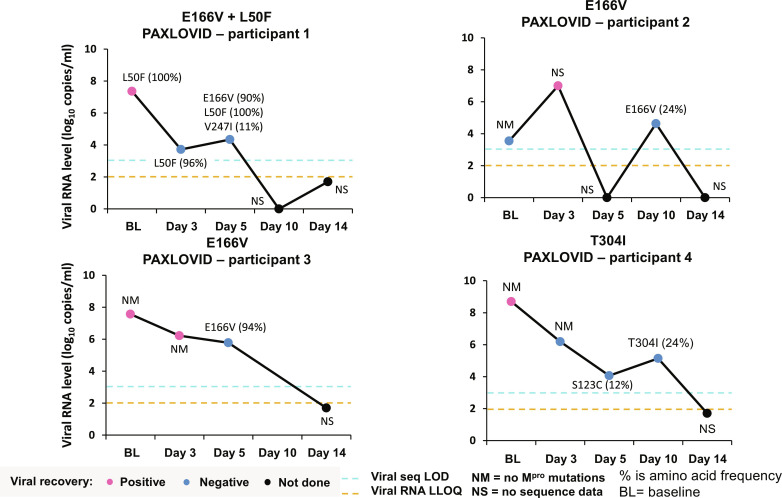
Patient viral load kinetic changes and time point at which the indicated mutations detected. Each graph depicts an individual patient virus load, the time point a mutation was detected and the frequency of the indicated mutation in the virus population. For any time point indicated as NS or not done, the viral load was insufficient for sequencing analysis.

At baseline, three individual mutations, L50F, P108S, and A191V were observed (Table 1). Each of these three mutations was part of specific composite mutation patterns associated with in vitro nirmatrelvir resistance identified in this study or previously (*22*–*24*). L50F was observed in one participant each in nirmatrelvir/ritonavir and placebo arm, P108S in one nirmatrelvir/ritonavir and three placebo participants, and A191V in two placebo participants. No composite M^pro^ mutations, or single mutations that conferred in vitro nirmatrelvir resistance pre-existed at baseline (Table 1), consistent with the surveillance finding of clinical isolates in GISAID database (Fig. 4). After baseline, E166V [up to 288-fold resistance to nirmatrelvir in vitro (*22*, *24*)] emerged in three Paxlovid-treated participants (Table 1 and Fig. 5), with participant 1 harboring the M^pro^ L50F mutation because baseline resulting in a L50F+E166V composite mutation at day 5. Participant 2 experienced viral RNA rebound at day 10 followed by virus clearance at day 14 (Fig. 5). All three participants were able to effectively control the virus by day 14, and none experienced COVID-19–related hospitalization or death from any cause through day 28 (primary endpoint of EPIC-HR). In addition, T304I emerged in one Paxlovid-treated participant at day 10 (Fig. 5, participant 4). The participant controlled the virus at day 14 without experiencing hospitalization or death. Notably, the nasal swab samples containing the E166V, L50+E166V and T304I mutations from the four Paxlovid-treated participants did not yield recoverable virus progenies in an in vitro virus recovery assay in VeroE6/TMPRSS2 cells (Fig. 5, A to D, blue dots). Their respective baseline samples produced viruses, except that of patient 2 from which day 3 virus without the E166V mutation was recovered (Fig. 5, A to D, pink dots). In summary, the identified M^pro^ resistance mutations were not associated with hospitalization or death in the EPIC-HR trial. Thus, their clinical relevance remains to be determined.

## DISCUSSION

In this study, SARS-CoV-2 resistance selection against nirmatrelvir in VeroE6-Pgp-KO cells led to the emergence of six single and combination mutations in M^pro^, which showed similar levels of EC_50_ values and fold changes in both VeroE6-Pgp-KO and A549-ACE2 cells. The T304I mutation appeared first in all selection schemes, followed by the addition of other mutations, indicating a stepwise resistance selection. Because Paxlovid dosing in the clinical EPIC-HR study led to median nirmatrelvir C_min_ plasma concentrations >5× WT EC_90_ or 14× EC_50_ in the physiologically relevant dNHBE cells (*7*, *9*), a <5× or <14× reduction in susceptibility to nirmatrelvir would theoretically retain clinical concentrations of nirmatrelvir above EC_90_ or EC_50_ for that mutant virus. While the single T304I and most of the double mutation patterns identified in this study exhibited low-level resistance, two specific mutation patterns A173V+T304I and T21I+S144A+T304I showed >20-fold resistance shift, and, therefore, should be closely monitored. There are other M^pro^ mutations such as E166V, L50F+E166V, and L50F+E166A+L167F identified in other laboratories that showed high levels of in vitro nirmatrelvir resistance, which also warrant close surveillance (*22*–*24*). The individual mutations, T21I, L50F, S144A, A173V, and T304I, identified in this study were also observed in vitro by other groups (*22*–*24*), confirming the reproducibility of the findings between different laboratories. These single mutations each conferred low level of resistance, i.e., T21I, L50F, S144A, A173V, and T304I, each led to 4.6-, 4.2-, 2.2-, 1.7-, and 5.5-fold resistance to nirmatrelvir (*24*). The A191V mutations which appeared transiently in passage 4 of scheme 5 in this study, as well as A173V were also detected following in vitro resistance selection versus boceprevir, a discontinued hepatitis C virus (HCV) protease inhibitor, and showed either no appreciable change (A191V) or low resistance (A173V) in vitro (*39*). Using a chimeric vesicular stomatitis virus (VSV) containing SARS-CoV-2 M^pro^ for resistance selection versus nirmatrelvir (*40*), a different set of mutations were observed that may or may not be specific for this artificial system. Using the site-directed mutagenesis using the same chimeric SARS-CoV-2 M^pro^/VSV system, a number of mutations were created among which several P168del-containing combination mutations showed different levels of in vitro nirmatrelvir resistance (*41*). The L167F mutation that alone led to a low level in vitro resistance to nirmatrelvir (*40*) was also identified in two other studies using SARS-CoV-2 (*23*, *24*), and again, A173V mutation was observed (*41*).

Notably, the two previously published in vitro nirmatrelvir resistance selection studies and the current study identified somewhat different sets of mutations in M^pro^ (*22*, *24*). For example, the abovementioned single mutations T21I, L50F, S144A, A173V, and T304I were each observed previously (*24*), but the composite mutation patterns that led to significant reduction in nirmatrelvir susceptibility, i.e., A173V+T304I and T21I+S144A+T304I are previously unidentified from this study. The previously identified E166V mutation that conferred by far the largest fold resistance in vitro (*22*, *24*) was not observed in this study. The reason for these differences in the selected resistance mutations could be due to the differences of the input virus quasi-species pool containing different mutations, differences in cell lines used, fitness of mutant virus (e.g., E166V alone), as well as differences in drug concentration and concentration escalations adopted (*22*, *24*). Also, some of the mutations (e.g., L50F) could function to offset the virus fitness deficit caused by the resistance mutation E166V (*22*). Regardless, these mutation patterns should be monitored in clinical studies and in circulating viruses. So far, the combination mutations with >5× in vitro EC_50_ increases identified in our in vitro studies are not represented in the GISAID database except for <10 instances of T21I+T304I of >13 million sequence entries analyzed (Fig. 4). None of these mutation patterns were detected in the EPIC-HR clinical study except for two individuals with E166V and one with L50F+E166V (Fig. 5 and Table 1). The previously described E166V and L50F+E166V mutations showed significant resistance in vitro (*22*, *24*) and that detection of E166V in Paxlovid-treated patients had been reported in two clinical case reports (*42*, *43*). However, none of the three patients harboring E166V or L50F+E166V experienced clinical treatment failure, and the counts of E166V virus remained very low pre- or post-Paxlovid EUA in GISAID (Fig. 4).

As Paxlovid becomes more widely used, monitoring the M^pro^ sequence mutations identified in this study as well as in other reports (*22*–*24*) will be important for clinical drug-resistant virus identification, infection management, and public health surveillance. Despite the millions of treatment courses of Paxlovid prescribed in the United States and globally, the frequency of in vitro nirmatrelvir resistance-associated mutations remains very low among the GISAID clinical isolates indicated in this study, as well as in other studies (*44*, *45*). This could be due to several mutually nonexclusive reasons: the binding mode of nirmatrelvir overlapping closely with the endogenous substrate, making any single M^pro^ mutation conferring significant resistance likely to reduce viral fitness; the high clinical concentrations of nirmatrelvir (*9*); the relatively low mutation rate of SARS-CoV-2 owing to the proofreading activity of a virus-encoded exonuclease (*46*, *47*); and the transient nature of most SARS-CoV-2 infections in patients (*48*, *49*).

## MATERIALS AND METHODS

### Cells

VeroE6-Pgp-KO, a VeroE6 cell line with P-gp multidrug transporter gene knockout cell line, was generated by Primary Pharmacology Group at Pfizer Research & Development in Groton, CT, USA (*25*). The cells were grown adherently in tissue culture–treated flasks and maintained in complete growth medium [Dulbecco’s modified Eagle’s medium (DMEM) supplemented with 1% antibiotic-antimycotic, 10 mM Hepes, and 10% fetal bovine serum (FBS)] with incubator settings at 37°C/5% CO_2_/95% humidity. VeroE6/TMPRSS2 cells were acquired from the JCRB Cell Bank (JCRB1819) and propagated in complete growth media consisting of DMEM supplemented with geneticin (1 mg/ml) and 10% FBS with incubators at 37°C and 5% CO_2_.

### SARS-CoV-2 virus

SARS-CoV-2 USA-WA1 strain was purchased from BEI Resources (NR-52281). Upon receipt, a virus stock was made in VeroE6/TEMPRSS2 cells. Briefly, VeroE6/TMPRSS2 cells were grown in T225 cm^2^ tissue culture flasks to approximately 80% confluency in cell maintenance media. On the day of infection, cell maintenance medium was removed and replaced with 10 ml of inoculum (viral growth medium containing 50 μl of SARS-CoV-2 USA-WA1 seed stock received from BEI Resources). The flask was incubated with gentle rocking 37°C/5% CO_2_/96% humidity for 20 min. After the incubation, 20 ml of additional viral growth medium was added, and the flask was returned to the incubator. Two days after infection, when all cells appeared to be infected, the media was harvested and clarified by centrifugation. The virus was aliquoted into 2-ml cryo-safe tubes and stored at 80°C.

This viral stock was used as parent virus (P0) for passaging.

### Plaque assay

In six-well plates, VeroE6-Pgp-KO cells were seeded to confluency. A 10-fold serial dilution of virus was performed, and cells were infected with 250 μl of each viral dilution for 1 hour at 37°C/5% CO_2_/95% humidity. After the 1-hour infection, a solid overlay of 1% SeaPlaque Agarose or semiliquid overlay consisting of 0.6% tragacanth gum in media was added to each well. After a 3-day infection, the overlay was removed, and the cell monolayers were fixed with 100% methanol for 15 min followed by staining with 0.5% crystal violet. Plaques were counted, and viral titer, expressed as plaque-forming units (PFU)/ml, was calculated.

### Median tissue culture infectious dose

A median tissue culture infectious dose (TCID_50_) assay was used to verify the viral titer of virus. VeroE6-Pgp-KO knockout cells were seeded into 96-well plates (2 × 10^4^ cells per well) and infected with a serially diluted virus. After 3-day incubation at 37°C/5% CO_2_/96% humidity plates were scored under the microscope for the absence or presence of CPE. The titer was calculated using the Spearman-Kärber method. The TCID_50_ can be converted to PFU by calculating the antilog of the TCID_50_ and multiplying by 0.7. This conversion was an estimate based on the rationale that the limiting dilution, which would infect 50% of the cell layers challenged, would be expected to produce a single plaque in a cell monolayer.

### CTG antiviral assay

Initially, the EC_50_ of nirmatrelvir was determined using the antiviral cytotoxicity assay. The assay is based on the measurement of CPE as quantified by cell adenosine triphosphate (ATP) levels in the presence of drug and virus using CellTiter-Glo (CTG) assay. The drug was serially diluted, from highest concentration to lowest, and VeroE6-Pgp-KO cells were infected with SARS-CoV-2 WA-1 strain at a MOI of 0.041. After a 3-day incubation, cell viability and the CPE is measured by the amount of ATP detected. EC_50_ was then calculated on the basis of % CPE at any given drug concentration against no virus control. The EC_50_ value was calculated by fitting the log(inhibitor concentration) versus CPE—Variable slope (four parameters) equation in GraphPad Prism software (version 9).

### Reverse transcription quantitative polymerase chain reaction–based antiviral assay

A reverse transcription quantitative polymerase chain reaction (RT-qPCR)–based assay was used to measure SARS-CoV-2 RNA following drug treatment and determine EC_50_ values. VeroE6-Pgp-KO cells were seeded into 96-well plates (2 × 10^4^ cells per well), mixed with a serial dilution of nirmatrelvir or remdesivir and infected with the parent or mutant virus at an MOI of 0.041. After a 2-day incubation, an in-plate cell lysis was performed followed by a heat step to inactivate the virus. A fixed portion of the lysate from each well was then combined with primers, probe and TaqPath master mix reagent, and virus genomic RNA were measured by RT-qPCR. Primers and probes targeting the *NSP10* gene in SARS-CoV-2 were Chinansp10MGB F: 5′-TGACCCTGTGGGTTTTACACTTAA-3′, Chinansp10MGB R: 5′-CAGCCATAACCTTTCCACATACC-3′, and Chinansp10MGB (probe): 5′-6FAMAACACAGTCTGTACCGTCTMGBNFQ-3′. Viral RNA copy number for each well was determined by the QuantStudio Design and Analysis software (Applied Biosystems) based on the serially diluted RNA amplicon standard curve that was run on each RT-qPCR plate. The RNA amplicon sequence was as follows: 5′GCUAAUGACCCUGUGGGUUUUACACUUAAAAACACAGUCUGUACCGUCUGCG GUAUGUGGAAAGGUUAUGGCUGUAGUU-3′.

Copy number values were used to calculate % inhibition of viral replication by nirmatrelvir in Excel using the calculation

$$\%\;\text{Inhibition}=100\%\times \frac{\text{No}\;\text{drug copy}\;\text{no}.-\text{sample copy}\;\text{no}.}{\text{No}\;\text{drug copy}\;\text{no}.}$$

Percent inhibition versus compound concentration was graphed in GraphPad Prism. EC_50_ values for each virus were calculated in GraphPad Prism using the log(inhibitor) versus response—variable slope (four parameters). The Hill slope was set to “must be less than 3.”

### Viral passaging in the presence of nirmatrelvir

SARS-CoV-2 USA-WA1 virus was serially passaged in Vero E6 Pgp knockout cells in the presence of nirmatrelvir starting at 1×EC_50_ and 1×EC_90_ (which equaled to 0.15 and 0.37 μM) as determined using the CTG antiviral assay. The initial passages were done at an MOI of 0.01 and at approximately MOI = 0.001 thereafter.

Several approaches were taken to successfully select mutant virus under drug pressure. Nirmatrelvir pressure was applied in several ways from the initial two passages. The passage which began at 1×EC_50_ was split into four different lineages.

In the first passaging scheme, nirmatrelvir was maintained at a constant concentration of 1×EC_50_ or 0.15 μM through nine passages. In the second passaging scheme, nirmatrelvir concentration was increased by 1×EC_50_ or 0.15 μM in each passage, reaching a final concentration of 1.31 μM by the final passage 9.

In the third passaging scheme, the nirmatrelvir concentration was increased by 1×EC_50_ or 0.15 μM through passage 3 reaching 3×EC_50_ or 0.34 μM. In passage 4, nirmatrelvir was doubled to 6×EC_50_ or 0.88 μM and was increased by 3×EC_50_ for every passage thereafter through passage 9 reaching a final concentration of 21×EC_50_ or 3.07 μM.

In the fourth passaging scheme, the nirmatrelvir concentration was increased by 1×EC_50_ or 0.15 μM through passage 3 reaching 3×EC_50_ or 0.34 μM. In passages 4 to 6, nirmatrelvir was increased by 3×EC_50_ reaching a concentration of 12×EC_50_ or 1.75 μM. In passages 7 and 8, the EC_50_ was doubled reaching 50×EC_50_ or 7.3 μM. The final passage 9 was done once again in 50×EC_50_. The fifth passaging scheme was the passage which began at 1×EC_90_ or 0.37 μM was passaged under constant 1×EC_90_ nirmatrelvir pressure through nine passages.

T25 cm^2^ tissue culture treated flasks seeded with Vero E6 Pgp KO cells were infected with 1 ml of inoculum consisting of drug and virus in viral growth media (VGM). After a 1-hour infection at 37°C/5% CO_2_/95% humidity, the inoculum was removed and washed with 1× PBS. Five milliliters of fresh VGM and compound was added to the flask and returned to 37°C, 5% CO_2_, 95% humidity. Infection could progress between 2 and 8 days for each passage, depending on amount of drug and viral fitness. If there was sufficient CPE at 2 days after infection (over 50%), the virus was harvested. If there was not sufficient CPE observed (less than 50%), the media was harvested, and replaced with fresh 5 ml of media containing drug, thus for some viruses we had a 2-day collection and a final collection. Only the final collection was used in subsequent passaging. If viral titer was not available from the previous passage, a blind passage was done, and MOI was back calculated.

When sufficient CPE was observed, viral growth medium from infected flasks was collected and clarified by centrifugation. Virus was aliquoted into 2-ml cryo-safe tubes and stored at −80°C. Viral titer was determined by TCID_50_ and converted in PFU/ml. Viral RNA was extracted for sequence determination from an aliquot taken at passages Pass #1 through Pass #9. A control viral passage was done with SARS-CoV-2 USA-WA1 passaged in 0.3% DMSO at MOI = 0.001 for 10 passages.

### Virus plaque purification

Plaque picking of SARS-CoV-2 USA-WA1 viral mutants was done from two lineages. The first from passaging scheme #3, sample from pass #7 passaged at 15×EC_50_ and the second from passaging scheme #5, samples from pass #4 (1×EC_90_) and pass #5 (1×EC_90_).

In a six-well plate, each virus was diluted to fewer than 10 plaques per well, well-spaced. The plaques were live stained with MTT and picked using a pipette tip. The plaque virus isolates were added to T25 cm^2^ tissue culture flasks which were preseeded with Vero E6 P-gp knockout cells. The plaques grew for 72 hours at 37°C/5%CO_2_/96% humidity. After sufficient CPE was observed, the virus was harvested and clarified by centrifugation. Supernatant was aliquoted and stored at −80°C.

A total of 88 plaques were picked and screened by Sanger sequencing to identify the substitutions. Six plaques were identified with the single, double, and triple mutations. These plaque picks were further amplified under nirmatrelvir pressure to prevent reversions. Each amplified plaque virus isolate was then tittered by TCID_50_ and deep sequenced. The six mutant viruses identified were single mutant T304I, double mutants T21I+T304I, L50F+T304I, T135I+T304I, and T304I+A173V, and triple mutant T21I+T304I+S144A.

### Sanger sequencing analysis

The nsp5 gene was amplified from extracted and purified SARS-CoV-2 RNA using the SuperScript III One-Step RT-PCR System with Platinum Taq High Fidelity DNA Polymerase (Invitrogen) and primers 9643 F nsp4 (5′-AGTGGATGGTTATGTTCACACCT-3′) and 11,141 R nsp6 (5′-CATTGCAAAAGCAGACATAGCA-3′). Amplified PCR products were purified with the ChargeSwitch PCR Clean-Up Kit (Invitrogen) and quantified via NanoDrop. Purified PCR products were sequenced with the BigDye Terminator v3.1 Cycle Sequencing Kit (Applied Biosystems) via the ABI 3730XL sequencer using the following primers: 10,032 F (5′-ATCACCTCAGCTGTTTTGCA-3′), 10,630 F (5′-CAGCTGGTACGGACACAACT-3′), 10,833 R (5′-CGGCAATTCCAGTTTGAGCA-3′), and 10,542 R (5′-GGTGCATGTAACAAAAAGAGACAC-3′). Sequencing files were aligned to the SARS-CoV-2 Washington strain WT sequence with the DNASTAR Lasergene 11 SeqMan Pro sequencing analysis program. All changes from the WT sequence were recorded.

### Amplicon-based SARS-CoV-2 next-generation library preparation and sequence determination

Nucleic acid purified from SARS-CoV-2 preparations is digested with deoxyribonuclease using the Invitrogen TURBO DNA-free Kit (cat. AM1907), followed by RNA purification using the Qiagen RNeasy MinElute Cleanup Kit (cat. 74204). Synthesis of cDNA is performed using random sequence primers with the Ampliseq cDNA Synthesis for Illumina kit (cat. 20022654).

The cDNA is used as template to specifically enrich for SARS-CoV-2 content in the sample by PCR. The xGen SARS-CoV-2 Amplicon Panel (IDT; cat. 10009832) is a single-pool design, containing overlapping primers to generate a total of 345 amplicons. PCR products range in size from 116 to 255 bp in length (average 150 bp) and cover >99% of the SARS-CoV-2 viral genome. Oligonucleotide primers directing the amplification of the viral amplicons were based upon available SARS-CoV-2 nucleotide sequences. Universal Next-Generation Sequencing Adaptors were ligated to the ends of the SARS-CoV-2 amplicons. The dual indexed amplicon libraries were purified with magnetic beads, normalized by manual pooling, and loaded to a flow cell for sequence determination (2 × 150 paired-end sequencing reads) using the Illumina NextSeq instrument (Illumina Inc.) according to the manufacturer’s instructions.

### Illumina bioinformatics workflow

A pair of FASTQ files for each sample containing sequence data from the clusters that pass filter is aligned to SARS-CoV-2 isolate Wuhan-Hu-1 reference genome (GenBank accession MN908947.3) using BWA. After initial alignment, primer sequences were trimmed using Sunspot trim primer function. At each nucleotide position, differences (threshold of >3% and coverage >30×) compared to the reference sequence were identified using Sunspot. Differences in nucleotide sequence that result in amino acid substitutions compared to the reference sequence were reported.

### Statistical analysis

The EC_50_ values of the viruses were analyzed with a one-way analysis of variance (ANOVA) (with mutant as the fixed effect), and Dunnett test was used to compare the EC_50_ of the mutant virus versus the aggregate or historic EC_50_ of the parent virus.

### M^pro^ protein production and purification

The M^pro^ mutant protein production and purification were the same as described previously (*31*). Briefly, site-directed mutagenesis was performed to engineer intended mutations into a WT SARS-CoV-2 M^pro^ construct (*50*) that contained an N-terminal PreScission protease cleavage site. Subsequent bacteria culture and protein purification procedures were performed as described previously (*31*).

### Enzyme characterization and kinetics

The enzymatic activity of the main protease, M^pro^ of SARS CoV-2 WT or variants was monitored using a continuous fluorescence resonance energy-transfer assay described previously (*31*). Briefly, WT or variant SARS CoV-2 M^pro^ activity was measured with a synthetic fluorogenic substrate peptide with the following sequence DABCYL-KTSAVLQ-SGFRKME-EDANS modeled on a consensus peptide. The assay reaction buffer contained 20 mM tris-HCl (pH 7.3), 100 nM NaCl, 1 mM EDTA, and 5 mM TCEP. For enzyme kinetic studies, SARS CoV-2 M^pro^ WT or variant was added to low-volume 384-well plates, and enzyme reactions were initiated with the addition of peptide substrate. Enzyme kinetic constants were calculated from initial rates. For enzyme inhibition studies, SARS CoV-2 M^pro^ WT or variant protease was added to low-volume 384-well plates containing compound dilutions and allowed to incubate for 20 min before the addition of peptide substrate to initiate the reaction. Enzyme activity was calculated from initial rates and expressed as percent activity based on control wells containing no compound and wells containing no enzyme. *K*_i_ values were fit to the Morrison equation with the enzyme concentration parameter allowed to float, the *K*_m_ parameter fixed to the substrate Km determined for each mutant, and the substrate concentration parameter fixed to 30 μM.

### Isothermal titration calorimetry

ITC was carried out using the PEAQ autoITC system from Malvern Inc. SARS-CoV-2 M^pro^ proteins were buffer exchanged into ITC buffer [25 mM tris-HCl (pH 8.0), 150 mM NaCl, 0.5 mM TCEP, and 1% DMSO] using Zeba spin desalting columns (Thermo, 7-kDa MWCO). The calorimetric cell containing SARS-CoV-2 M^pro^ protein (WT or mutants) was titrated with nirmatrelvir dissolved in the same buffer [25 mM tris-HCl (pH 8.0), 150 mM NaCl, 0.5 mM TCEP, and 1% DMSO]. One-microliter aliquots of nirmatrelvir at a concentration of 100 μM in the syringe was injected into the cell containing 10 μM SARS-CoV-2 M^pro^ at 2-min intervals. The heat evolved upon each injection of ligand was obtained from the integral of the calorimetric signal. The heat associated with the binding of protease inhibitor to protein was obtained by subtracting the heat of dilution from the heat of reaction. All measurements were made at 25°C and done at least in triplicate. Data were fit to a one-site binding model using Malvern’s MicroCal PEAQ-ITC Analysis Software (version 1.4). Statistical analysis and plotting of *K*_D_, ΔH, and −*T*Δ*S* were done in GraphPad Prism (version 9).

### Crystallization and structure determination

Crystals used to determine the structures of SARS-CoV-2 M^pro^ mutants were obtained from apo protein (T21I+T304I; L50F+T304I) or by cocrystallization with nirmatrelvir (A173V+T304I; T21I+S144A+T304I) or by soaking nirmatrelvir into pregrown apo crystals (S144A; A173V). All crystals were obtained via vapor diffusion in sitting drops using MRC-Maxi (Swissci) plates, where proteins at 6.00 to 7.40 mg/ml were mixed 1:1 (300 nl + 300 nl) with well solution (see specific details below). Plates were incubated at 21°C, and crystals typically grew within 5 days. All crystals were passed through a cryoprotectant consisting of their respective well buffers containing 20% ethylene glycol and flash cooled in liquid nitrogen in preparation for data collection.

*Crystallization of SARS-CoV-2 M^pro^ T21I*+*T304I and L50F*+*T304I*: Apo crystals of both double mutants were obtained under similar conditions. Protein (6.00 and 6.60 mg/ml, respectively) was set up for crystallization with wells consisting of 0.1 M MES (pH 5.63 to 6.00, respectively), and 13% PEG 4000.

*Cocrystallization of SARS-CoV-2 M^pro^ A173V*+*T304I*: SARS-CoV-2 M^pro^ A173V+T304I protein (7.40 mg/ml) was incubated with 2.0 mM nirmatrelvir (in 100% DMSO) for a molar ratio of approximately 1:9, for 18 hours at 4°C. The complex was then set up for crystallization with well buffer containing 5.0% 2-methyl-2,4-penanediol, 0.1 M Hepes (pH 7.50) and 10.0% w/v PEG 10000.

*Cocrystallization of SARS-CoV-2 M^pro^ T21I*+*S144A*+*T304I*: SARS-CoV-2 M^pro^ triple mutant (7.40 mg/ml) was incubated with 2.0 mM nirmatrelvir (in 100% DMSO) for a molar ratio of approximately 1:9, for 18 hours at 4°C. The complex was then set up for crystallization with well buffer consisting of 20.0% v/v 2-propanol, 0.1 M tris (pH 8.00), and 5.0% w/v PEG 8000.

*Crystallization of SARS-CoV-2 M^pro^ S144A*: Crystals of SARS-CoV-2 M^pro^ S144A in complex with nirmatrelvir were obtained via soaking. Pregrown apo crystals of the mutant were first prepared by setting up protein at 7.3 mg/ml with well solution consisting of 0.1 M imidazole (pH 7.00) and 20.0% w/v PEG 6000. Nirmatrelvir (1 mM final concentration) was then added directly to crystallization drops and allowed to soak into crystals for 3 hours at 21°C.

*Crystallization of SARS-CoV-2 M^pro^ A173V*: Crystals of SARS-CoV-2 M^pro^ A173V in complex with nirmatrelvir were prepared by soaking. Pregrown apo crystals of the mutant were first prepared by setting up protein at 7.4 mg/ml with well solution consisting of 0.1 M Hepes (pH 7.00), 20.0% w/v PEG 6000 and 0.2 M sodium chloride. Nirmatrelvir (1 mM final concentration) was then added to crystallization drops and soaked into crystals for 3 hours at 21°C.

X-ray diffraction data were collected for all samples except the triple mutant (T21I+S144A+T304I) at −173°C at IMCA-CAT 17-ID beamline of the Advanced Photon Source (APS) at Argonne National Labs, a U.S. Department of Energy (DOE) Office of Science User Facility operated for the DOE Office of Science using the Eiger 2 x 9 M detector (Dectris) and wavelength of 1.0 Å. Use of the IMCA-CAT beamline 17-ID (or 17-BM) at the Advanced Photon Source was supported by the companies of the Industrial Macromolecular Crystallography Association through a contract with Hauptman-Woodward Medical Research Institute. Data for T21I+S144A+T304I were collected at beamline CMCF-ID at the Canadian Light Source with the Eiger 2 x 9 M detector (Dectris).

The structures were determined by difference Fourier [autoBUSTER (*51*)] or molecular replacement [DIMPLE (*52*)] and refined using the anisotropically scaled data as described previously for WT SARS-CoV-2-M^pro^ in complex with nirmatrelvir (*7*). Diffraction data processing and model refinement statistics for each of the mutant M^pro^ are provided in table S2.

### Molecular dynamics simulations

All the molecular dynamic simulations in the study were carried out in the following procedure. Pre-equilibrated TIP3P water molecules were added around protein/ligand or protein/peptide complex. The total simulation box is of 103 × 62 × 74 Å^3^, allowing for approximately 10 Å between the box edge and the protein or lipid. We added three sodium ions to neutralize the system. The final simulation box contained 36,363 atoms for ligand bound enzyme complex and 37,656 atoms for peptide-bound enzyme complex. We carried out MD simulations using with the program openMM (*53*) using with the Amber ff14SB force field proteins (*54*). Small-molecule Parameters for the nirmatrelvir were assigned on the basis of GAFF2 (*55*) together with am1bbc partial charges. The simulation box was first minimized by 5000 steps and equilibrated by NPT simulation using periodic boundary conditions at 300 K in 250 K steps every picosecond with a 2-fs step size. All the atoms were free to move in the equilibrium. Constant temperature control was achieved with Langevin dynamics with 5 ps − 1 damping. Pressure was held at 1 atm by a Nose–Hoover Langevin piston with a decay period of 100 fs and a damping time of 50 fs. Particle mesh Ewald summation was used to compute the long-range electrostatic interaction for the periodic cells. After equilibration, 500 ns simulation run was collected for the complex analysis.

The starting coordinates of the SARS-CoV-2 M^pro^/nirmatrelvir complex was from PDB 7RFS (*7*). The related covalent bond parameters between Cys145 and nirmatrelvir were defined in AmberTool (*56*). The starting coordinates of the enzyme mutants were generated by the “protein mutation” function in MOE [Molecular Operating Environment (MOE), version 2022.02, Chemical Computing Group]. The same protein and ligand force field parameters and preparation were applied for the molecular dynamic simulation of the enzyme mutant/nirmatrelvir complexes.

The pdb structure of M^pro^-nsp5/nsp6, 7T8M (*33*), was used in the MD simulation. Both the M^pro^ T304 and the substrate peptide nsp5/nsp6 P3 Threonine were mutated to Isoleusine as the comparor mutant complex. The Vdw and electrostatic interaction were calculated between the M^pro^ protein and the peptide P4-P1, Val-Thr(Ile)-Phe-Gln, with 80 Å as the threshold to define the calculation pairs. Dielectric constant ε 20 was applied in the electrostatic interaction (*57*, *58*) calculation.

### M^pro^ small-molecule inhibitor and substrate peptide similarity calculation

Substrate peptide and M^pro^ complex structure:

nsp4/nsp5: 7DVP

nsp5/nsp6: 7DVW

nsp6/nsp7: 7DVX

Inhibitors’ structure:

nirmatrelvir: 7RFS

Shionogi: 7VU6

PF-0085231: 6XHM

FitTversky Shape Overlap Score is the percentage of the overlapped (AՈB) portion out of the total reference molecule’s (inhibitor’s) shape: AՈB/B, where A is the shape of substrate peptide (reference molecule), B is the shape of inhibitor (Fit molecule).

Adjusted overlap score was calculated after both comparison molecules’ shape was adjusted by solvent exposed area. Both A and B shape were adjusted by how much it is exposed to the solvent. When an atom’s solvent accessible area (SAS) change is less than 90%, it means that this atom remains largely solvent exposed upon the molecule binding to the protein pocket. Therefore, that atom’s radius is adjusted by 50% in the shape calculation. For atom i, if 1 − SASi complex / SAS i, ligand <0.1; then Radius i = 0.5*Radius i. The surface area, shape and overlap scores were calculated using the Openeye Scientific OEShape toolkit (version 3.6.0.0, Candance Molecular Science).

### M^pro^ mutation analysis from GISAID SARS-CoV-2 genomes

Genome sequences and patient metadata for SARS-CoV-2 isolates were obtained from GISAID (epicov.org) database. Incomplete genomes of less than 29,000 nucleotides and/or > 5% Ns were excluded. AA mutations in M^pro^ were previously annotated by GISAID using CoVsurver (corona.bii.a-star.edu.sg/faq.html) and provided with metadata for each isolate.

### SARS-CoV-2 sequencing and genotyping of clinical samples

SARS-CoV-2 genomes from clinical nasal swab samples were sequenced using the SWIFT biosciences next-generation amplicon-based method (University of Washington Medicine Clinical Virology Laboratory). Virus from baseline [day 1 (D1)], D3, D5, D10, and D14 participant samples that meet the sequencing limit of detection (LoD) (viral concentrations ≥2.70 log_10_ copies/ml) were sequenced and genotyped by mapping against the reference sequence (NCBI: NC_045512.2). To reduce sequencing artifacts, data from local (unvalidated) swabs and swabs with viral RNA level <3.0 log_10_ copies/ml were excluded from analysis. In addition, to ensure quality control of sequence data, the genome acceptability criteria were as defined as positive controls reported with 1 million raw reads, >85% identity to the reference sequence (NCBI: NC_045512.2), 75× average genome coverage, 1000× mean M^pro^ gene coverage, >90% of M^pro^ at 100× or higher, and < 10% Ns in the consensus genome.

### Calling a mutation and emergent mutation

The AA sequence was compared to the reference Wuhan-Hu-1 strain (*59*) and M^pro^ mutations were called for any residue with ≥10% AA frequency (AAFREQ).

Any distinct mutation was called emergent if the mutation was absent (AA frequency (AAFREQ) <10% or not called) for the participant in their baseline sample, but the same mutation was present (AAFREQ ≥10%) for the same participant in any postbaseline sample (D3, D5, D10, or D14). Mutations resulting in frameshift were excluded from analysis. For day 1 visit, only results within 1 hour post start of dosing were treated as baseline (D1) data.

### Infectious clinical sample virus recovery assay

Virus recovery assay was carried out by a contract research organization (CRO), Microbiologics (San Diego, CA), who were blinded to the treatment arms of the samples tested. Briefly, 1 day before infection, VeroE6/TMPRSS2 cells were seeded with 3 × 10^5^ cells per well in 12-well plates and incubated overnight for 18 to 24 hours at 37°C and 5% CO_2_. Before infection, the wells were confirmed to be approximately 80 to 90% confluent. Samples were thawed to room temperature and then diluted 1:4, 1:10, 1:100 and 1:1000 in Inoculation Media (DMEM, 5% FBS and 1× Antibiotic-Antimycotic). Before infection, the media was aspirated, and the cells were washed with 1 ml of DPBS. Each diluted sample (150 μl; one replicate per dilution) was added to the cells and incubated for 1 hour at 37°C and 5% CO_2_. After 1 hour, the sample was removed and replaced with 1 ml of Inoculation Media. The plates were incubated for 5 days at 37°C and 5% CO_2_. After 5 days, the wells were scored for the presence or absence of infection. Virus-containing media was recovered and stored at −80°C from wells that were positive for infection.

### Ethics statement

Clinical NP samples were obtained from participants in the EPIC-HR trial (*9*); each clinical site was approved by an ethics committee, and all participants provided written informed consent.

## Supplementary Materials

### This PDF file includes:

Figs. S1 and S2 Tables S1 to S3 References
