# Supplementary material for: In vitro selection and analysis of SARS-CoV-2 nirmatrelvir resistance mutations contributing to clinical virus resistance surveillance
Source: Sci Adv. 2024 Jul 24;10(30):eadl4013. doi: 10.1126/sciadv.adl4013 (PMC11268423; doi:10.1126/sciadv.adl4013)
Supplement: Supplementary file 1 — Figs. S1 and S2 Tables S1 to S3 References [file sciadv.adl4013_sm.pdf]

Science Advances
AAAS

Supplementary Materials for

In vitro selection and analysis of SARS-CoV-2 nirmatrelvir resistance mutations contributing to clinical virus resistance surveillance

Yuao Zhu *et al.*

Corresponding author: Rhonda D. Cardin, rhonda.cardin@pfizer.com

Sci. Adv. **10**, eadl4013 (2024)
DOI: 10.1126/sciadv.adl4013

This PDF file includes:

Figs. S1 and S2
Tables S1 to S3
References

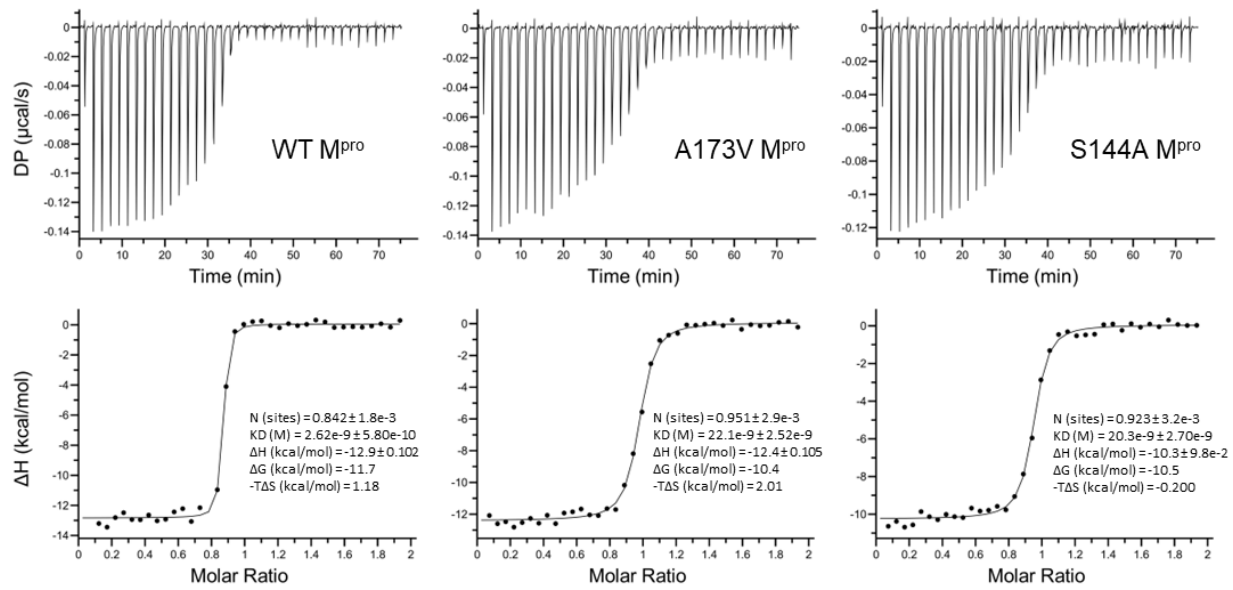

Fig. S1. Isothermal titration calorimetry of purified M^{pro} WT and mutants. Representative thermographs for WT (left), A173V (middle), and S144A (right) M^{pro} variants. Fitted thermodynamic parameters of binding using a one-site model are shown for each.

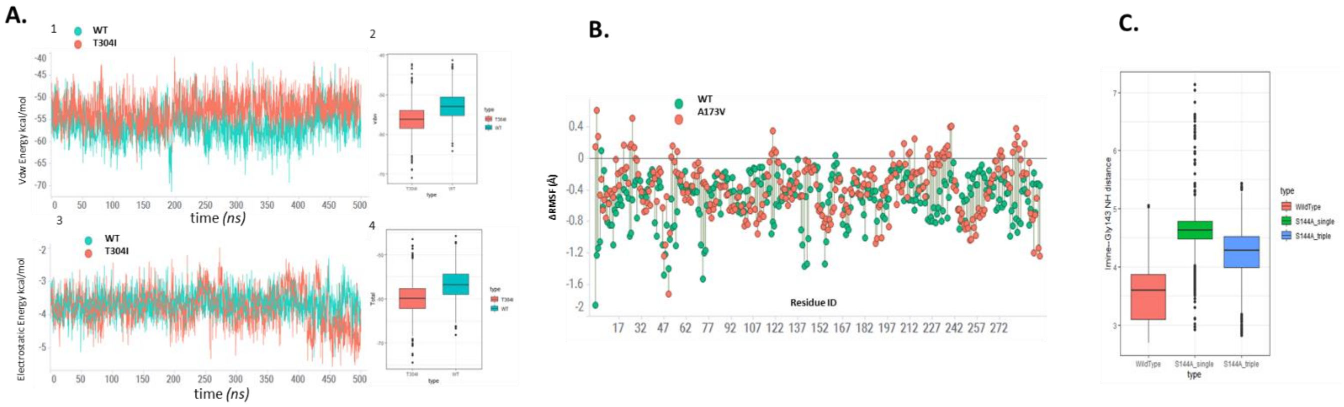

Fig. S2. Molecular dynamic simulation. A: (1-4) MD simulation of M^{pro} complex with nsp5/nsp6 peptide substrate (pdb, 7T8M) and mutant substrate peptide with T304I. Time course pairwise (1) Van der Waal and (3) electrostatic energy between protein and substrate peptide. The corresponding boxplots of the pairwise energy distribution is displayed in boxplot (2) Van der Waal and (4) Total energy, the sum of electrostatic and vdw, in kcal/mol. **B**: The calculated Root Mean Squared Fluctuation (RMSF) difference between bound complex and apo by residues from MD simulation. RMSF difference in WT is colored in green and RMSF difference in A173V single mutation is in red. **C**: MD simulation comparison of M^{pro} WT, S144A and T304I+T21I+S144A in the presence of nirmatrelvir. Boxplot of the distance distribution between inhibitor adduct imine nitrogen to Gly143 backbone NH nitrogen from Molecular Dynamic simulation.

Table S1. Amino acid (AA) substitutions detected within ORFs other than M^{pro}

ORF	SNV	AA substitution	T304I (%)	T304I+ L50F (%)	T304I+ T21I (%)	T304I+ T135I (%)	T304I+ T21I+ S144A (%)	T304I+ A173V (%)
**nsp2**	C1938T	Ser378Phe		15.7				
	G2585A	Val594Ile	61.4					
**nsp3**	C6633T	Ala1305Val			8.7			
	T7348G	Asn1543Lys						100
**nsp6**	A11462G	Met164Val						65.5
	T11552G	Phe184Val		13.1				
**nsp10**	A13131G	Gln36Arg			6.6			
**nsp12**	A14754C	Lys438Asn			9.4			
**nsp13**	A16518C	Lys94Asn						100
	C16799T	Thr188Ile	24.8	17.7				
	T16913C	Val266Ala						
	C17321T	Ala362Val			10.7			
	C17639T	Ser468Leu	100	100	100	100	100	
**nsp14**	A18298G	Ile87Val	33.9	19.8	100		100	
	A18911G	Asp291Gly					100	
**S**	C21762T	Ala67Val						11.8
	C24237T	Ala892Val					24.7	
**orf3a**	A25501G	Ile37Val	100	100	100	100	100	
**M**	C26895T	His125Tyr		5.9				
**Orf6**	C27208T	His3Tyr						4.3
	C27223T	Gln8*	37.5	22.1				
**orf7a**	C27473T	Thr27Ile						11.8

Table S2. Data Statistics for X-ray diffraction results

**Mutant**	**S144A**	**A173V**	**A173V +T304I**	**T21I+ S144A + T304I**	**T21I + T304I**	**L50F + T304I**
PDB entry ID	9AUJ	9AUK	9AUL	9AUM	9AUN	9AUO
Ligand	Nirmatrelvir	Nirmatrelvir	Nirmatrelvir	Nirmatrelvir	APO	APO
Resolution	47.94 – 1.49	112.67-1.88	105.48-2.42	41.68-1.54	68.32-2.29	68.43-2.42
Space group	C2	$P2_1$	$P2_12_12$	$P2_12_12$	C2	C2
Unit cell dimensions [Å]	a = 114.6, b = 53.1, c = 45.5	a = 45.5, b = 54.6, c = 114.9	a = 45.6, b = 64.1, c = 105.5	a = 45.4, b = 63.9, c = 105.2	a = 123.3, b = 82.1, c = 64.4	a = 121.9, b = 82.7, c = 64.2
Unit cell dimensions [º]	$\alpha = \gamma$ =90.0, β = 102.7	$\alpha = \gamma$ =90.0, β = 101.4	$\alpha = \beta = \gamma$ =90.0	$\alpha = \beta = \gamma$ =90.0	$\alpha = \gamma$ =90.0, β = 90.3	$\alpha = \gamma$ =90.0, β = 90.4
Total number of reflections*	122868 (5176)	109217 (5786)	66601 (2616)	199415 (9582)	73190 (3514)	71925 (3601)
Unique reflections*	36035 (1802)	31343 (1568)	10221 (511)	36169 (1808)	20879 (1044)	20317 (1016)
Multiplicity*	3.4 (2.9)	3.5 (3.7)	6.5 (5.1)	5.5 (5.3)	3.5 (3.4)	3.5 (3.5)
Completeness (%), spherical*	81.9 (27.3)	69.4 (14.8)	82.8 (25.1)	78.4 (21.1)	72.0 (17.9)	83.4 (22.5)
Completeness (%), ellipsoidal*	85.5 (37.9)	90.8 (46.9)	93.6 (63.7)	93.0 (64.6)	84.8 (45.0)	91.5 (42.8)
Mean $I/\sigma(I)$*	13.3 (1.3)	7.8 (1.6)	6.1 (1.2)	9.8 (1.6)	14.4 (1.4)	8.9 (1.2)
R_{merge}†	0.039 (0.664)	0.104 (0.848)	0.259 (1.340)	0.106 (1.097)	0.043 (0.806)	0.044 (0.807)
R_{pim}‡	0.025 (0.450)	0.066 (0.518)	0.109 (0.644)	0.049 (0.509)	0.027 (0.520)	0.037 (0.641)
$CC_{1/2}$§	0.999 (0.632)	0.996 (0.592)	0.986 (0.507)	0.998 (0.581)	0.999 (0.524)	0.998 (0.578)
**Refinement Statistics**						
Reflections used	36035	31343	10221	36162	20871	20317
Reflections used for R_{free}	1740	1553	490	1816	1052	1036
R_{cryst} ¶	0.201	0.207	0.203	0.195	0.242	0.276
R_{free}#	0.219	0.253	0.277	0.229	0.291	0.315
**Ramachandran Plot**						

Favored regions (%)	99.0	98.8	96.3	97.3	95.0	95.4
Allowed Regions (%)	0.7	0.8	3.3	2.3	4.8	4.3
Outlier regions (%)	0.3	0.3	0.3	0.3	0.2	0.3

* Numbers in parentheses refer to the highest resolution shell

† $R_{merge}= \sum_{hkl} \sum_{i=1}^{n} | I_i(hkl) - \bar{I}(hkl) | / \sum_{hkl} \sum_{i=1}^{n} I_i\ (hkl)$

‡ $R_{pim}= \sum_{hkl} \sqrt{1/(n-1)} \sum_{i=1}^{n} | I_i(hkl) - \bar{I}(hkl) / \sum_{hkl} \sum_{i=1}^{n} I_i\ (hkl)$ (*60*)

§ $CC_{1/2}$ = xxx as defined by Karplus and Diederichs (*61*)

¶ $R_{cryst}= \sum_{hkl} | F_o(hkl) - F_c(hkl) | \sum_{hkl} | F_o(hkl) |$, where F_o and F_c are the observed and calculated structure factors, respectively.

R_{free} is the same as R_{cryst}, but for 5% of the data randomly omitted from refinement (*62*)

Table S3. Tversky shape overlap between substrate peptide and M^{pro} inhibitors

Substrate peptide	active-site peptide sequence*	Nirmatrelvir		S-217622		PF-00835231	
		Adjusted Overlap†	Overlap‡	Adjusted overlap	Overlap	Adjusted overlap	Overlap
nsp4/nsp5	SA**VL**Q-S	**91.40%**	74.60%	65.10%	52.20%	84.70%	74.20%
nsp5/nsp6	GV**TF**Q-S	86.60%	72.90%	61.90%	50.20%	69.60%	62.20%
nsp6/nsp7	VA**TV**Q-S	87.40%	71.70%	61.30%	49.10%	87.50%	76.80%

* peptide amino acids included in the shape comparison. P2 and P3 amino acids are highlighted in bold

† FitTversky shape overlap score adjusted by solvent exposed area

‡ FitTversky shape overlap score by two molecules without adjustment

REFERENCES AND NOTES

1. R. Lu, X. Zhao, J. Li, P. Niu, B. Yang, H. Wu, W. Wang, H. Song, B. Huang, N. Zhu, Y. Bi, X. Ma, F. Zhan, L. Wang, T. Hu, H. Zhou, Z. Hu, W. Zhou, L. Zhao, J. Chen, Y. Meng, J. Wang, Y. Lin, J. Yuan, Z. Xie, J. Ma, W. J. Liu, D. Wang, W. Xu, E. C. Holmes, G. F. Gao, G. Wu, W. Chen, W. Shi, W. Tan, Genomic characterisation and epidemiology of 2019 novel coronavirus: implications for virus origins and receptor binding. *Lancet* **395**, 565–574 (2020).

2. L. Zhang, D. Lin, X. Sun, U. Curth, C. Drosten, L. Sauerhering, S. Becker, K. Rox, R. Hilgenfeld, Crystal structure of SARS-CoV-2 main protease provides a basis for design of improved α-ketoamide inhibitors. *Science* **368**, 409–412 (2020).

3. Z. Jin, X. du, Y. Xu, Y. Deng, M. Liu, Y. Zhao, B. Zhang, X. Li, L. Zhang, C. Peng, Y. Duan, J. Yu, L. Wang, K. Yang, F. Liu, R. Jiang, X. Yang, T. You, X. Liu, X. Yang, F. Bai, H. Liu, X. Liu, L. W. Guddat, W. Xu, G. Xiao, C. Qin, Z. Shi, H. Jiang, Z. Rao, H. Yang, Structure of Mpro from SARS-CoV-2 and discovery of its inhibitors. *Nature* **582**, 289–293 (2020).

4. J. Qiao, Y.S. Li, R. Zeng, F.L. Liu, R.H. Luo, C. Huang, Y.F. Wang, J. Zhang, B. Quan, C. Shen, X. Mao, X. Liu, W. Sun, W. Yang, X. Ni, K. Wang, L. Xu, Z.L. Duan, Q.C. Zou, H.L. Zhang, W. Qu, Y.H.P. Long, M.H. Li, R.C. Yang, X. Liu, J. You, Y. Zhou, R. Yao, W.P. Li, J.M. Liu, P. Chen, Y. Liu, G.F. Lin, X. Yang, J. Zou, L. Li, Y. Hu, G.W. Lu, W.M. Li, Y.Q. Wei, Y.T. Zheng, J. Lei, S. Yang, SARS-CoV-2 M^{pro} inhibitors with antiviral activity in a transgenic mouse model. *Science* **371**, 1374–1378 (2021).

5. K. Anand, J. Ziebuhr, P. Wadhwani, J. R. Mesters, R. Hilgenfeld, Coronavirus main proteinase (3CLpro) structure: basis for design of anti-SARS drugs. *Science* **300**, 1763–1767 (2003).

6. T. Pillaiyar, M. Manickam, V. Namasivayam, Y. Hayashi, S. H. Jung, An overview of severe acute respiratory syndrome-coronavirus (SARS-CoV) 3CL protease inhibitors: Peptidomimetics and small molecule chemotherapy. *J. Med. Chem.* **59**, 6595–6628 (2016).

7. D. R. Owen, C. M. N. Allerton, A. S. Anderson, L. Aschenbrenner, M. Avery, S. Berritt, B. Boras, R. D. Cardin, A. Carlo, K. J. Coffman, A. Dantonio, L. di, H. Eng, R.A. Ferre, K. S. Gajiwala, S. A. Gibson, S. E. Greasley, B. L. Hurst, E. P. Kadar, A. S. Kalgutkar, J. C. Lee, J. Lee, W. Liu, S. W. Mason, S.

Noell, J. J. Novak, R. S. Obach, K. Ogilvie, N. C. Patel, M. Pettersson, D. K. Rai, M. R. Reese, M. F. Sammons, J. G. Sathish, R. S. P. Singh, C. M. Steppan, A. E. Stewart, J. B. Tuttle, L. Updyke, P. R. Verhoest, L. Wei, Q. Yang, Y. Zhu, An oral SARS-CoV-2 M(pro) inhibitor clinical candidate for the treatment of COVID-19. *Science* **374**, 1586–1593 (2021).

8. FDA, Prescribing Information: Paxlovid™ [U.S. Food and Drug Administration (FDA), 2023], pp. 1–34; www.accessdata.fda.gov/drugsatfda_docs/label/2023/217188s000lbl.pdf.

9. J. Hammond, H. Leister-Tebbe, A. Gardner, P. Abreu, W. Bao, W. Wisemandle, M. Baniecki, V.M. Hendrick, B. Damle, A. Simón-Campos, R. Pypstra, J.M. Rusnak, EPIC-HR Investigators, Oral nirmatrelvir for high-risk, nonhospitalized adults with Covid-19. *N. Engl. J. Med.* **386**, 1397–1408 (2022).

10. J. A. Lewnard, J. M. McLaughlin, D. Malden, V. Hong, L. Puzniak, B. K. Ackerson, B. J. Lewin, J. S. Kim, S. F. Shaw, H. Takhar, L. Jodar, S. Y. Tartof, Effectiveness of nirmatrelvir–ritonavir in preventing hospital admissions and deaths in people with COVID-19: A cohort study in a large US health-care system. *Lancet Infect. Dis.* **23**, 806–815 (2023).

11. R. Arbel, Y. Wolff Sagy, M. Hoshen, E. Battat, G. Lavie, R. Sergienko, M. Friger, J. G. Waxman, N. Dagan, R. Balicer, Y. Ben-Shlomo, A. Peretz, S. Yaron, D. Serby, A. Hammerman, D. Netzer, Nirmatrelvir use and severe Covid-19 outcomes during the Omicron surge. *N. Engl. J. Med.* **387**, 790–798 (2022).

12. C. K. H. Wong, I.C.H. Au, K.T.K. Lau, E.H.Y. Lau, B.J. Cowling, G.M. Leung, Real-world effectiveness of molnupiravir and nirmatrelvir plus ritonavir against mortality, hospitalisation, and in-hospital outcomes among community-dwelling, ambulatory patients with confirmed SARS-CoV-2 infection during the omicron wave in Hong Kong: An observational study. *Lancet* **400**, 1213–1222 (2022).

13. S. Mason, J. P. Devincenzo, S. Toovey, J. Z. Wu, R. J. Whitley, Comparison of antiviral resistance across acute and chronic viral infections. *Antiviral Res.* **158**, 103–112 (2018).

14. L. Menéndez-Arias, R. Delgado, Update and latest advances in antiretroviral therapy. *Trends Pharmacol. Sci.* **43**, 16–29 (2022).

15. R. J. Whitley, C. A. Boucher, B. Lina, J. S. Nguyen-van-Tam, A. Osterhaus, M. Schutten, A. S. Monto, Global assessment of resistance to neuraminidase inhibitors, 2008–2011: The Influenza Resistance Information Study (IRIS). *Clin. Infect. Dis.* **56**, 1197–1205 (2013).

16. F. Zoulim, S. Locarnini, Hepatitis B virus resistance to nucleos(t)ide analogues. *Gastroenterology* **137**, 1593–1608.e2 (2009).

17. C. Sarrazin, S. Zeuzem, Resistance to direct antiviral agents in patients with hepatitis C virus infection. *Gastroenterology* **138**, 447–462 (2010).

18. L. J. Stevens, A. J. Pruijssers, H. W. Lee, C. J. Gordon, E. P. Tchesnokov, J. Gribble, A. S. George, T. M. Hughes, X. Lu, J. Li, J. K. Perry, D. P. Porter, T. Cihlar, T. P. Sheahan, R. S. Baric, M. Götte, M. R. Denison, Mutations in the SARS-CoV-2 RNA-dependent RNA polymerase confer resistance to remdesivir by distinct mechanisms. *Sci. Transl. Med.* **14**, eabo0718 (2022).

19. S. Gandhi, J. Klein, A. J. Robertson, M. A. Peña-Hernández, M. J. Lin, P. Roychoudhury, P. Lu, J. Fournier, D. Ferguson, S. A. K. Mohamed Bakhash, M. Catherine Muenker, A. Srivathsan, E. A. Wunder Jr, N. Kerantzas, W. Wang, B. Lindenbach, A. Pyle, C. B. Wilen, O. Ogbuagu, A. L. Greninger, A. Iwasaki, W. L. Schulz, A. I. Ko, De novo emergence of a remdesivir resistance mutation during treatment of persistent SARS-CoV-2 infection in an immunocompromised patient: A case report. *Nat. Commun.* **13**, 1547 (2022).

20. NIH, Gain of Function Research [National Institutes of Health (NIH) Office of Science Policy 2023]; https://osp.od.nih.gov/policies/national-science-advisory-board-for-biosecurity-nsabb/gain-of-function-research/.

21. FDA, COVID-19: Developing Drugs and Biological Products for Treatment or Prevention: Guidance for Industry [U.S. Food and Drug Adminstration (FDA), 2021)], pp. 1–22; www.fda.gov/media/167274/download.

22. Y. Zhou, K. A. Gammeltoft, L. A. Ryberg, L. V. Pham, H. D. Tjørnelund, A. Binderup, C. R. Duarte Hernandez, C. Fernandez-Antunez, A. Offersgaard, U. Fahnøe, G. H. J. Peters, S. Ramirez, J. Bukh, J. M. Gottwein, Nirmatrelvir-resistant SARS-CoV-2 variants with high fitness in an infectious cell culture system. *Sci. Adv.* **8**, eadd7197 (2022).

23. D. Jochmans, C. Liu, K. Donckers, A. Stoycheva, S. Boland, S. K. Stevens, C. de Vita, B. Vanmechelen, P. Maes, B. Trüeb, N. Ebert, V. Thiel, S. de Jonghe, L. Vangeel, D. Bardiot, A. Jekle, L. M. Blatt, L. Beigelman, J. A. Symons, P. Raboisson, P. Chaltin, A. Marchand, J. Neyts, J. Deval, K. Vandyck, The substitutions L50F, E166A, and L167F in SARS-CoV-2 3CLpro are selected by a protease inhibitor in vitro and confer resistance to nirmatrelvir. *MBio* **14**, e0281522 (2023).

24. S. Iketani, H. Mohri, B. Culbertson, S. J. Hong, Y. Duan, M. I. Luck, M. K. Annavajhala, Y. Guo, Z. Sheng, A.C. Uhlemann, S. P. Goff, Y. Sabo, H. Yang, A. Chavez, D. D. Ho, Multiple pathways for SARS-CoV-2 resistance to nirmatrelvir. *Nature* **613**, 558–564 (2023).

25. Y. Zhu, J. Binder, I. Yurgelonis, D. K. Rai, S. Lazarro, C. Costales, K. Kobylarz, P. McMonagle, C. M. Steppan, L. Aschenbrenner, A. S. Anderson, R. D. Cardin, Generation of a VeroE6 Pgp gene knock out cell line and its use in SARS-CoV-2 antiviral study. *Antiviral Res.* **208**, 105429 (2022).

26. A. Molla, M. Korneyeva, Q. Gao, S. Vasavanonda, P. J. Schipper, H.M. Mo, M. Markowitz, T. Chernyavskiy, P. Niu, N. Lyons, A. Hsu, G. R. Granneman, D. D. Ho, C. A.B. Boucher, J. M. Leonard, D. W. Norbeck, D. J. Kempf, Ordered accumulation of mutations in HIV protease confers resistance to ritonavir. *Nat. Med.* **2**, 760–766 (1996).

27. C. Sui, T. Xiao, S. Zhang, H. Zeng, Y. Zheng, B. Liu, G. Xu, C. Gao, Z. Zhang, SARS-CoV-2 NSP13 inhibits type I IFN production by degradation of TBK1 via p62-dependent selective autophagy. *J. Immunol.* **208**, 753–761 (2022).

28. S. Y. Fung, K.L. Siu, H. Lin, C.P. Chan, M. L. Yeung, D.Y. Jin, SARS-CoV-2 NSP13 helicase suppresses interferon signaling by perturbing JAK1 phosphorylation of STAT1. *Cell Biosci.* **12**, 36 (2022).

29. W. Lu, B.J. Zheng, K. Xu, W. Schwarz, L. du, C. K. L. Wong, J. Chen, S. Duan, V. Deubel, B. Sun, Severe acute respiratory syndrome-associated coronavirus 3a protein forms an ion channel and modulates virus release. *Proc. Natl. Acad. Sci. U.S.A.* **103**, 12540–12545 (2006).

30. Y. M. Baez-Santos, S. E. St John, A. D. Mesecar, The SARS-coronavirus papain-like protease: Structure, function and inhibition by designed antiviral compounds. *Antiviral Res.* **115**, 21–38 (2015).

31. S. E. Greasley, S. Noell, O. Plotnikova, R.A. Ferre, W. Liu, B. Bolanos, K. Fennell, J. Nicki, T. Craig, Y. Zhu, A. E. Stewart, C. M. Steppan, Structural basis for the in vitro efficacy of nirmatrelvir against SARS-CoV-2 variants. *J. Biol. Chem.* **298**, 101972 (2022).

32. M. Bastos, O. Abian, C. M. Johnson, F. Ferreira-da-Silva, S. Vega, A. Jimenez-Alesanco, D. Ortega-Alarcon, A. Velazquez-Campoy, Isothermal titration calorimetry. *Nat. Rev.Methods Prim.* **3**, 17 (2023).

33. A. M. Shaqra, S. N. Zvornicanin, Q. Y. J. Huang, G. J. Lockbaum, M. Knapp, L. Tandeske, D. T. Bakan, J. Flynn, D. N. A. Bolon, S. Moquin, D. Dovala, N. Kurt Yilmaz, C. A. Schiffer, Defining the substrate envelope of SARS-CoV-2 main protease to predict and avoid drug resistance. *Nat. Commun.* **13**, 3556 (2022).

34. J. Řezáč, Non-covalent interactions atlas benchmark data sets: Hydrogen bonding. *J. Chem. Theory Comput.* **16**, 2355–2368 (2020).

35. H. Mitsuya, K. Maeda, D. Das, A. K. Ghosh, Development of protease inhibitors and the fight with drug-resistant HIV-1 variants. *Adv. Pharmacol.* **56**, 169–197 (2008).

36. B. Boras, R. M. Jones, B. J. Anson, D. Arenson, L. Aschenbrenner, M. A. Bakowski, N. Beutler, J. Binder, E. Chen, H. Eng, H. Hammond, J. Hammond, R. E. Haupt, R. Hoffman, E. P. Kadar, R. Kania, E. Kimoto, M. G. Kirkpatrick, L. Lanyon, E. K. Lendy, J. R. Lillis, J. Logue, S. A. Luthra, C. Ma, S. W. Mason, M. E. McGrath, S. Noell, R. S. Obach, M. N. O' Brien, R. O'Connor, K. Ogilvie, D. Owen, M. Pettersson, M. R. Reese, T. F. Rogers, R. Rosales, M. I. Rossulek, J. G. Sathish, N. Shirai, C. Steppan, M. Ticehurst, L. W. Updyke, S. Weston, Y. Zhu, K. M. White, A. García-Sastre, J. Wang, A. K. Chatterjee, A. D. Mesecar, M. B. Frieman, A. S. Anderson, C. Allerton, Preclinical characterization of

an intravenous coronavirus 3CL protease inhibitor for the potential treatment of COVID19. *Nat. Commun.* **12**, 6055 (2021).

37. Y. Unoh, S. Uehara, K. Nakahara, H. Nobori, Y. Yamatsu, S. Yamamoto, Y. Maruyama, Y. Taoda, K. Kasamatsu, T. Suto, K. Kouki, A. Nakahashi, S. Kawashima, T. Sanaki, S. Toba, K. Uemura, T. Mizutare, S. Ando, M. Sasaki, Y. Orba, H. Sawa, A. Sato, T. Sato, T. Kato, Y. Tachibana, Discovery of S-217622, a noncovalent oral SARS-CoV-2 3CL protease inhibitor clinical candidate for treating COVID-19. *J. Med. Chem.* **65**, 6499–6512 (2022).

38. S. Elbe, G. Buckland-Merrett, Data, disease and diplomacy: GISAID's innovative contribution to global health. *Glob Chall* **1**, 33–46 (2017).

39. K. A. Gammeltoft, Y. Zhou, L. A. Ryberg, L. V. Pham, A. Binderup, C. R. D. Hernandez, A. Offersgaard, U. Fahnøe, G. H. J. Peters, S. Ramirez, J. Bukh, J. M. Gottwein, Substitutions in SARS-CoV-2 Mpro selected by protease inhibitor boceprevir confer resistance to nirmatrelvir. *Viruses* **15**, (2023).

40. E. Heilmann, F. Costacurta, S. A. Moghadasi, C. Ye, M. Pavan, D. Bassani, A. Volland, C. Ascher, A. K. H. Weiss, D. Bante, R. S. Harris, S. Moro, B. Rupp, L. Martinez-Sobrido, D. von Laer, SARS-CoV-2 3CL(pro) mutations selected in a VSV-based system confer resistance to nirmatrelvir, ensitrelvir, and GC376. *Sci. Transl. Med.* **15**, eabq7360 (2023).

41. S. A. Moghadasi, E. Heilmann, A. M. Khalil, C. Nnabuife, F. L. Kearns, C. Ye, S. N. Moraes, F. Costacurta, M. A. Esler, H. Aihara, D. von Laer, L. Martinez-Sobrido, T. Palzkill, R. E. Amaro, R. S. Harris, Transmissible SARS-CoV-2 variants with resistance to clinical protease inhibitors. *Sci. Adv.* **9**, eade8778 (2023).

42. Y. Hirotsu, H. Kobayashi, Y. Kakizaki, A. Saito, T. Tsutsui, M. Kawaguchi, S. Shimamura, K. Hata, S. Hanawa, J. Toyama, Y. Miyashita, M. Omata, Multidrug-resistant mutations to antiviral and antibody therapy in an immunocompromised patient infected with SARS-CoV-2. *Med* **4**, 813–824.e4 (2023).

43. N. S. Zuckerman, E. Bucris, D. Keidar-Friedman, M. Amsalem, T. Brosh-Nissimov, Nirmatrelvir resistance-de novo E166V/L50V mutations in an immunocompromised patient treated with prolonged

nirmatrelvir/ritonavir monotherapy leading to clinical and virological treatment failure—A case report. *Clin. Infect. Dis.* **78**, 352–355 (2024).

44. J. T. Lee, Q. Yang, A. Gribenko, B. S. Perrin Jr, Y. Zhu, R. Cardin, P. A. Liberator, A. S. Anderson, L. Hao, Genetic surveillance of SARS-CoV-2 M^{pro} reveals high sequence and structural conservation prior to the introduction of protease inhibitor Paxlovid. *MBio* **13**, e0086922 (2022).

45. J. D. Ip, A. Wing-Ho Chu, W.M. Chan, R. Cheuk-Ying Leung, S. M. Umer Abdullah, Y. Sun, K. K.W. To, Global prevalence of SARS-CoV-2 3CL protease mutations associated with nirmatrelvir or ensitrelvir resistance. *EBioMedicine* **91**, 104559 (2023).

46. E. Minskaia, T. Hertzig, A. E. Gorbalenya, V. Campanacci, C. Cambillau, B. Canard, J. Ziebuhr, Discovery of an RNA virus 3′ ->5′ exoribonuclease that is critically involved in coronavirus RNA synthesis. *Proc. Natl. Acad. Sci. U.S.A.* **103**, 5108–5113 (2006).

47. E. C. Smith, H. Blanc, M. C. Surdel, M. Vignuzzi, M. R. Denison, Coronaviruses lacking exoribonuclease activity are susceptible to lethal mutagenesis: Evidence for proofreading and potential therapeutics. *PLOS Pathog.* **9**, e1003565 (2013).

48. B. Hu, H. Guo, P. Zhou, Z. L. Shi, Characteristics of SARS-CoV-2 and COVID-19. *Nat. Rev. Microbiol.* **19**, 141–154 (2021).

49. S. Lee, T. Kim, E. Lee, C. Lee, H. Kim, H. Rhee, S. Y. Park, H.J. Son, S. Yu, J. W. Park, E. J. Choo, S. Park, M. Loeb, T. H. Kim, Clinical course and molecular viral shedding among asymptomatic and symptomatic patients with SARS-CoV-2 infection in a community treatment center in the Republic of Korea. *JAMA Intern. Med.* **180**, 1447–1452 (2020).

50. H. X. Su, S. Yao, W.F. Zhao, M.J. Li, J. Liu, W.J. Shang, H. Xie, C.Q. Ke, H.C. Hu, M.N. Gao, K.Q. Yu, H. Liu, J.S. Shen, W. Tang, L.K. Zhang, G.F. Xiao, L. Ni, D.W. Wang, J.P. Zuo, H.L. Jiang, F. Bai, Y. Wu, Y. Ye, Y.C. Xu, Anti-SARS-CoV-2 activities in vitro of Shuanghuanglian preparations and bioactive ingredients. *Acta Pharmacol. Sin.* **41**, 1167–1177 (2020).

51. C. Vonrhein, C. Flensburg, P. Keller, A. Sharff, O. Smart, W. Paciorek, T. Womack, G. Bricogne, Data processing and analysis with the autoPROC toolbox. *Acta Crystallogr. D Biol. Crystallogr.* **67**, 293–302 (2011).

52. Collaborative Computational Project, Number 4, The CCP4 suite: Programs for protein crystallography. *Acta Crystallogr. D Biol. Crystallogr.* **50**, 760–763 (1994).

53. P. Eastman, J. Swails, J. D. Chodera, R. T. McGibbon, Y. Zhao, K. A. Beauchamp, L.P. Wang, A. C. Simmonett, M. P. Harrigan, C. D. Stern, R. P. Wiewiora, B. R. Brooks, V. S. Pande, OpenMM 7: Rapid development of high performance algorithms for molecular dynamics. *PLoS Comput. Biol.* **13**, e1005659 (2017).

54. J. A. Maier, C. Martinez, K. Kasavajhala, L. Wickstrom, K. E. Hauser, C. Simmerling, ff14SB: Improving the accuracy of protein side chain and backbone parameters from ff99SB. *J. Chem. Theory Comput.* **11**, 3696–3713 (2015).

55. D. Vassetti, M. Pagliai, P. Procacci, Assessment of GAFF2 and OPLS-AA general force fields in combination with the water models TIP3P, SPCE, and OPC3 for the solvation free energy of druglike organic molecules. *J. Chem. Theory Comput.* **15**, 1983–1995 (2019).

56. J. Wang, R. M. Wolf, J. W. Caldwell, P. A. Kollman, D. A. Case, Development and testing of a general amber force field. *J. Comput. Chem.* **25**, 1157–1174 (2004).

57. L. Li, C. Li, Z. Zhang, E. Alexov, On the dielectric "constant" of proteins: Smooth dielectric function for macromolecular modeling and its implementation in DelPhi. *J. Chem. Theory Comput.* **9**, 2126–2136 (2013).

58. M. Amin, J. Kupper, Variations in proteins dielectric constants. *ChemistryOpen* **9**, 691–694 (2020).

59. F. Wu, S. Zhao, B. Yu, Y.M. Chen, W. Wang, Z.G. Song, Y. Hu, Z.W. Tao, J.H. Tian, Y.Y. Pei, M.L. Yuan, Y.L. Zhang, F.H. Dai, Y. Liu, Q.M. Wang, J.J. Zheng, L. Xu, E. C. Holmes, Y.Z. Zhang, A new coronavirus associated with human respiratory disease in China. *Nature* **579**, 265–269 (2020).

60. M. S. Weiss, R. Hilgenfeld, On the use of the merging R factor as a quality indicator for X-ray data. *J. Appl. Cryst.* **30**, 203–205 (1997).

61. P. A. Karplus, K. Diederichs, Linking crystallographic model and data quality. *Science* **336**, 1030–1033 (2012).

62. A. T. Brünger, C.W. Carter Jr., R.M. Sweet, in *Methods in Enzymology* (Academic Press, 1997), vol. **277**, pp. 366–396.
